# Associations Between Adolescent Psychological Factors and Physical Activity and Sedentary Behavior in Young Adulthood by Disability Status

**DOI:** 10.3390/healthcare14213733

**Published:** 2026-11-03

**Authors:** Seungyeon Park, Jihoon Jung

**Affiliations:** 1 Department of Health, Physical Education & Exercise Science, Norfolk State University, Norfolk, VA 23504, USA; 2 Department of Geography and the Environment, University of North Texas, Denton, TX 76203, USA; jihoon.jung@unt.edu

**Keywords:** physical activity, sedentary behavior, psychological factors, disability status, adolescence, young adulthood

## Abstract

**Objective:** Physical activity (PA) during adolescence plays a crucial role in shaping lifelong health behaviors; however, longitudinal evidence on the associations between psychological factors and PA and sedentary behavior (SB) remains limited, particularly among populations defined by disability status. This study examined associations between adolescent depressive symptoms, parental control, parental warmth, and self-esteem and changes in PA and SB from adolescence to young adulthood among individuals with and without physical disabilities. **Methods:** Using nationally representative data from the National Longitudinal Study of Adolescent Health (N = 7339), including 427 participants classified as having a physical disability, we examined changes in PA and SB from adolescence to young adulthood. We separately developed 16 survey-weighted multinomial logistic regression models representing combinations of activity type (PA or SB), psychological factor (depressive symptoms, parental warmth, parental control, or self-esteem), and disability status (yes or no), while adjusting for demographic and socioeconomic characteristics. **Results:** Several psychological factors were associated with longitudinal changes in PA and SB. Among participants with physical disabilities, higher normalized self-esteem scores were associated with a greater likelihood of decreased PA (RRR = 15.89, 95% CI: 2.11–119.53). Higher normalized depressive symptom scores were associated with both a greater likelihood of decreased SB (RRR = 19.08, 95% CI: 1.12–326.40) and increased SB (RRR = 10.30, 95% CI: 1.19–89.07), relative to maintaining stable levels. **Conclusions:** Adolescent psychological and parental factors were associated with longitudinal patterns of PA and SB from adolescence to young adulthood. These findings provide evidence of associations within both disability groups and support further research examining how psychological factors relate to changes in PA and SB across the transition to young adulthood.

## 1. Introduction

Sedentary behavior (SB) is a major public health concern across all age groups, potentially leading to increased health disparities and risks of health conditions such as overweight, obesity, and chronic diseases including diabetes, cardiovascular disease, cancer and mental health disorders like depression [1,2]. On the other hand, engaging in physical activity (PA) provides numerous health benefits, significantly enhancing both physical and mental well-being. Regular PA strengthens the immune system [3], prevents acute and chronic diseases [4], and improves cognitive function [5] and mental health [6,7]. Studies have also shown that individuals who frequently participate in PA experience better stress management [8], increased workplace productivity [9], and strengthened social connections [10], which in turn promotes emotional well-being [11] and interpersonal skills [12].

PA is particularly important for children and adolescents, as being active during these formative years can greatly influence their long-term habits and commitment to a healthy lifestyle [13,14,15]. Studies have shown that low levels of PA in childhood and adolescence often persist into adulthood, highlighting the need for early and sustained engagement in PA [16]. Despite its benefits, most children and adolescents fail to meet the current Centers for Disease Control and Prevention recommendation of at least 60 min of moderate to vigorous physical activity per day (MVPA) [17]. The situation is even more concerning for children with disabilities, who tend to participate in significantly lower levels of PA compared to their peers without disabilities [18].

To tackle low level of PA and high level of SB among children and adolescents with disabilities, it is essential to identify relevant determinants that correlate with their PA and SB levels in order to develop effective intervention strategies [19]. Previous studies have successfully identified various demographic (e.g., age, gender, race/ethnicity), socioeconomic (e.g., income, employment), and environmental factors (e.g., access to facilities, neighborhood safety) that significantly influence their PA and SB levels [20,21,22,23].

Importantly, PA participation among individuals with disabilities should not be understood solely in terms of individual characteristics. Psychological factors operate within broader social and environmental contexts, including accessibility, opportunities for participation, social support, and structural barriers that may facilitate or constrain PA engagement [24]. Although the present study focuses specifically on psychological factors, these factors should therefore be considered within this broader context. However, there is limited research specifically investigating longitudinal associations between psychological factors in adolescence and changes in PA and SB. Most previous studies have examined the general associations between PA/SB and psychological factors using cross-sectional designs, rather than longitudinal approaches that capture changes across this developmental period. Among the psychological and interpersonal factors potentially relevant to PA and SB, depressive symptoms and self-esteem represent individual psychological characteristics, whereas parental warmth and parental control reflect aspects of the family environment that may be associated with adolescents’ health behaviors. These factors were therefore examined to better understand how adolescent psychological and parental characteristics are associated with subsequent patterns of PA and SB.

The present study examined longitudinal associations between adolescent psychological factors, including depressive symptoms, parental control, parental warmth, and self-esteem, and changes in PA and SB during the transition from adolescence to young adulthood among individuals with and without physical disabilities. Specifically, we examined whether each of these psychological factors was associated with patterns of decrease, stability, or increase in PA and SB over time within each disability group. Given the exploratory, research-question-driven nature of this secondary analysis, the study was not designed to test directional hypotheses derived from a specific theoretical framework. Rather, the analyses were conducted to characterize longitudinal associations between these adolescent psychological factors and subsequent patterns of PA and PI separately among individuals with and without physical disabilities.

## 2. Materials and Methods

This study was approved by the Norfolk State University Institutional Review Board.

The National Longitudinal Study of Adolescent to Adult Health (Add Health) is a nationally representative longitudinal study that has followed participants from adolescence into adulthood. Data were collected across multiple waves, beginning with Wave I during adolescence and followed by subsequent assessments across Waves II–IV. The present study used data specifically from Waves I and IV to examine longitudinal changes and associations between adolescent characteristics and health-related outcomes in adulthood.

### 2.1. Physical Disability

Physical disability status assessed at Wave I was used as the baseline grouping variable for the present longitudinal analysis. Wave I includes a range of disability-related questions addressing functional difficulties, use of assistive equipment, need for personal-care assistance, and perceptions of disability. It contains two components: an In-Home Questionnaire completed by an adolescent and a Parent Survey completed by a parent, each of which collects the information separately.

We individually identified adolescents as having a disability or not using the In-Home Questionnaire and the Parent Survey in Wave I. In the In-Home Questionnaire, adolescents were classified as having a disability if they provided a positive response to any of the questions assessing: (1) limb difficulties, use of assistive equipment, or need for personal-care assistance; (2) self-perception of disability or being viewed as disabled by others; (3) difficulties with walking, standing, reaching, grasping, or holding objects; and (4) blindness or deafness. In the Parent Survey, adolescents were also classified as having a disability if their parents provided a positive response to any of the questions regarding: (1) limb difficulties, use of assistive equipment, or need for personal-care assistance and (2) perceiving the adolescent as disabled or being viewed as such by others. These indicators were used to identify participants with a physical disability at baseline rather than to distinguish among specific disability diagnoses or types. This classification was based on a previously established method that converts disability severity scores into a binary classification (with or without a disability) [25]. To provide greater transparency regarding the operationalization of physical disability, the specific indicators used to derive the classification are described above rather than relying solely on the previously published classification procedure.

Agreement between adolescent and parent reports was also examined. Of the 15,701 cases, 14,860 (94.6%) showed agreement, with adolescents and parents reporting the same disability score. In 677 cases (4.3%), adolescents reported higher disability scores than their parents, whereas in 164 cases (1.0%), parents reported higher disability scores than adolescents.

### 2.2. PA and SB Measures

We extracted PA and SB measures from each wave and calculated the differences between the two time points to determine whether participants’ PA and SB levels increased, remained stable, or decreased over time. For PA levels, we used the total bouts of MVPA per week, drawn from a 1-week recall questionnaire. In Wave I, we selected questions relevant to MVPA activities including inline skating, skateboarding, bicycling, football, basketball, baseball, soccer, softball, swimming, jogging, walking, martial arts, jump roping, dancing, and gymnastics. In Wave IV, we used a similar set of questions from Wave I. The only difference in questions between the two periods was that, in Wave IV, questions were divided into more specific categories based on activity types, unlike the broader grouping used in Wave I. The primary difference relevant to the present analysis was that Wave I grouped multiple types of activities within broader activity questions, whereas Wave IV assessed activities using more specific activity categories. In other words, the level of detail in the activity questions differed between the two waves, although comparable types of physical activities were represented in both assessments. For SB, we measured the total weekly time spent on selected leisure-time behaviors assessed in Add Health, including watching television, viewing movies, playing computer games, and listening to the radio.

Because the same questionnaires for PA and SB were not asked in each wave, we compared the relative differences in PA and SB between Waves I and IV rather than using direct comparisons [26]. For this, we first summed the total frequency of PA and the total time of SB separately for Waves I and IV to measure the overall levels of PA and SB at the individual level. We then normalized the sum of PA and SB using min-max normalization (Equation (1)) for each wave.

(1) $$x_{\mathrm{normalized}}=\frac{\left(x-x_{\min}\right)}{\left(x_{\max}-x_{\min}\right)}$$

where x_normalized_ is the normalized value; x is the initial value; x_max_ and x_min_ are the maximum and minimum value. Then, we calculated the difference in normalized PA/SB between waves. Next, we categorized the differences into three groups based on standard deviation (SD) to avoid issues with misclassification due to small variations: decrease (<−1 SD), stable (−1 SD~1 SD), and increase (>1 SD). Descriptive summaries of the original, non-normalized PA and SB variables by wave among participants with and without disabilities are provided in Supplementary Tables S1 and S2.

### 2.3. Depressive Symptoms

A total of 19 questions from Wave I were used to measure depressive symptoms (Supplementary Table S3). These questions were selected based on the Center for Epidemiological Studies Depression (CES-D) scale [27], which has been widely used in multiple studies to understand depressive symptomatology in the general population [28,29,30]. Responses were scaled from 0 (never or rarely) to 3 (most of the time or all of the time). Among the 19 questions, 4 were reverse-coded: (1) You felt that you were just as good as other people, (2) You felt hopeful about the future, (3) You were happy, and (4) You enjoyed life. Consequently, higher scores indicate higher levels of depressive symptoms. We then summed the scores across all 19 questions to represent individuals’ levels of depressive symptoms and standardized the resulting score to facilitate comparison with other psychological factors.

### 2.4. Parental Control and Warmth

Based on previous studies we selected seven questions to measure parental control [31,32,33] and six questions to assess parental warmth [34,35,36] from Wave I (Supplementary Tables S4 and S5). Parental control questions include whether parents allow children to make their own decisions about activities, behavioral habits, and preferences, such as watching TV, spending time with friends, going to bed, and choosing what to wear. Responses to these seven questions were scored either no (0) or yes (1), with higher scores indicating a higher level of parental control. On the other hand, parental warmth includes questions about children’s perceptions of their mother’s and father’s attitudes toward them, such as how close they feel to their parents (1: not at all–5: very much), how much they believe their parents care about them (1: not at all–5: very much), and how often they feel their parents are warm and loving toward you (1: strongly agree–5: strongly disagree). We reverse-coded two questions (Most of the time, your mother/father is warm and loving toward you.) to ensure that higher scores indicate higher levels of parental warmth. Finally, we summed the corresponding items to create composite scores and then standardized the scores to facilitate comparison of coefficients across psychological factors.

### 2.5. Self-Esteem

To measure self-esteem, we selected four questions (Supplementary Table S6) from Wave I, asking how the child feels about themselves [34,37,38]. The questions include (1) “you have a lot of good qualities,” (2) “you have a lot to be proud of,” (3) “you like yourself just the way you are,” and (4) “you feel like you are doing everything just about right.” These questions were scored from 1 (strongly agree) to 5 (strongly disagree). We reverse-coded the items so that lower scores indicate lower self-esteem and higher scores indicate higher self-esteem. All the answers were then summed and standardized.

### 2.6. Demographic and Socioeconomic Status

To control for the impact of demographic and socioeconomic factors, we selected multiple variables commonly identified as influencing PA and PI levels [20,21,22,23]. These variables include race, ethnicity, age, gender, education, and income. Race was categorized into three groups: White, Black or African American, and others. Ethnicity was classified as Hispanic or non-Hispanic, gender as male or female, and education as with or without a high school diploma. These data were derived from Wave IV to control for participants’ current socioeconomic status.

### 2.7. Data Analysis: Survey-Weighted Multinomial Logistic Regression

We used survey-weighted multinomial logistic regression, which accounts for the complex sampling design of Add Health, to investigate how psychological factors during adolescence are associated with PA and SB patterns in young adulthood using the same individuals after controlling for multiple demographic and socioeconomic variables. Models were estimated using the svy_vglm function in the *svyVGAM* and *survey* package in r(version 4.6.0), which accounts for the complex survey design, including primary sampling units, strata, and sampling weights. For this analysis, we used the Wave 4 weight [39]. Diagnostic considerations included the absence of problematic multicollinearity among predictors and adequate observations across outcome categories for stable parameter estimation.

This method uses maximum likelihood estimation to predict the probability that a case falls into one of several outcome categories based on one or more independent variables. Unlike other multivariate methods, it does not require assumptions such as normality, linear relationships, or homoscedasticity. Results are typically reported as relative risk ratios (RRRs), which compare the probability of belonging to a particular category relative to a reference category. An RRR greater than 1 indicates higher probability of the outcome compared to the reference category, whereas an RRR less than 1 indicates lower probability.

In this study, we first checked for multicollinearity among the independent variables to prevent inflated or inaccurate coefficient estimates, and found no violations (all variance inflation factors were close to 1.0). We also calculated the correlations among the four psychological factors and added the correlation matrix (Supplementary Table S7). We then conducted complementary mutually adjusted models in which all four psychological factors were included simultaneously. The results of these analyses are reported in Supplementary Tables S8–S11.

In the next step, we created two sets of survey-weighted multinomial logistic regressions using changes in PA and SB levels between Waves I and IV as the dependent variables. For each outcome (PA or SB), we created eight separate models, representing every combination of psychological factor (depressive symptoms, parental control, parental warmth, and self-esteem) and disability status (no disability, any disability). This resulted in a total of sixteen models across PA and SB. In the model, we standardized the psychological factors using Equation (1) to ensure that all values fall within the same range of 0 to 1. The independent variables in each model included one psychological factor, along with demographic variables and socioeconomic variables. Each model included one psychological factor as the independent variable and was estimated separately by disability status. We did not apply a formal correction for multiple comparisons because the analyses were based on pre-specified research questions and were intended to characterize patterns of associations across psychological factors rather than to test a single primary hypothesis. Given the smaller sample size of the disability subgroup, applying a stringent correction could also substantially reduce statistical power.

## 3. Results

### 3.1. Descriptive Summary

The survey-weighted descriptive analysis showed that the mean age of participants was 15.07 years (95% CI: 14.85–15.29). Most participants were White (74.9%, 95% CI: 70.0–79.8%), followed by Black participants (15.8%, 95% CI: 11.8–19.8%). The sample was evenly distributed by sex, with 50.0% male (95% CI: 48.7–51.3%) and 50.0% female (95% CI: 48.7–51.3%). The average income was $46.1K (95% CI: $42.6–$49.5K). Regarding ethnicity, 88.0% were non-Hispanic (95% CI: 84.6–91.3%), while 12.0% were Hispanic (95% CI: 8.7–15.4%). Most participants completed high school, accounting for 92.7% of the sample (95% CI: 91.4–94.0%).

Descriptive statistics were estimated using survey-weighted methods. Weighted proportions for categorical variables were estimated using the *svymean* function from the *survey* package in R(version 4.6.0), which provides population-representative estimates and appropriate variance estimation. The initial sample consisted of 15,701 participants who were 12–18 years of age at Wave I and 24–34 years of age at Wave IV. After excluding participants without valid sampling weights, 9421 participants remained. Complete-case analysis was then applied by excluding participants with missing values for the variables included in each analysis. The final analytic sample consisted of 7339 participants for the PA analyses (6912 without disabilities and 427 with disabilities) and 7285 participants for the SB analyses (6864 without disabilities and 421 with disabilities) (Supplementary Figure S1).

Results showed that the majority (94.4%) did not have a disability, while the remaining respondents (5.6%) had a disability (Table 1). Differences in normalized PA and SB across waves were recategorized into three groups to examine how PA and SB changed over time: decrease, stable, and increase. Approximately 25% were classified as decreasing their PA, 15% as increasing, and 60% as remaining stable, in both groups. In contrast, about 10% were classified as either decreasing or increasing SB, with 80% remaining stable in both groups. These differences in proportions were due to the distinct shapes of their distributions.

We also statistically examined the differences in normalized PA and SB score changes between Wave IV and Wave I among participants with and without disabilities using a survey-weighted linear regression model. The analysis accounted for the Add Health complex survey design by incorporating the primary sampling unit, sampling strata, and survey weights. Disability status was included as the independent variable, and normalized PA and SB score changes were treated as continuous dependent variables. The survey-weighted analysis showed a significant difference in normalized PA score changes between participants with and without disabilities (*p* = 0.012). Participants with disabilities had, on average, 0.015 points higher normalized PA score changes than those without disabilities. However, no significant difference was found in normalized SB score changes between the two groups (*p* = 0.964).

**Figure 1:**
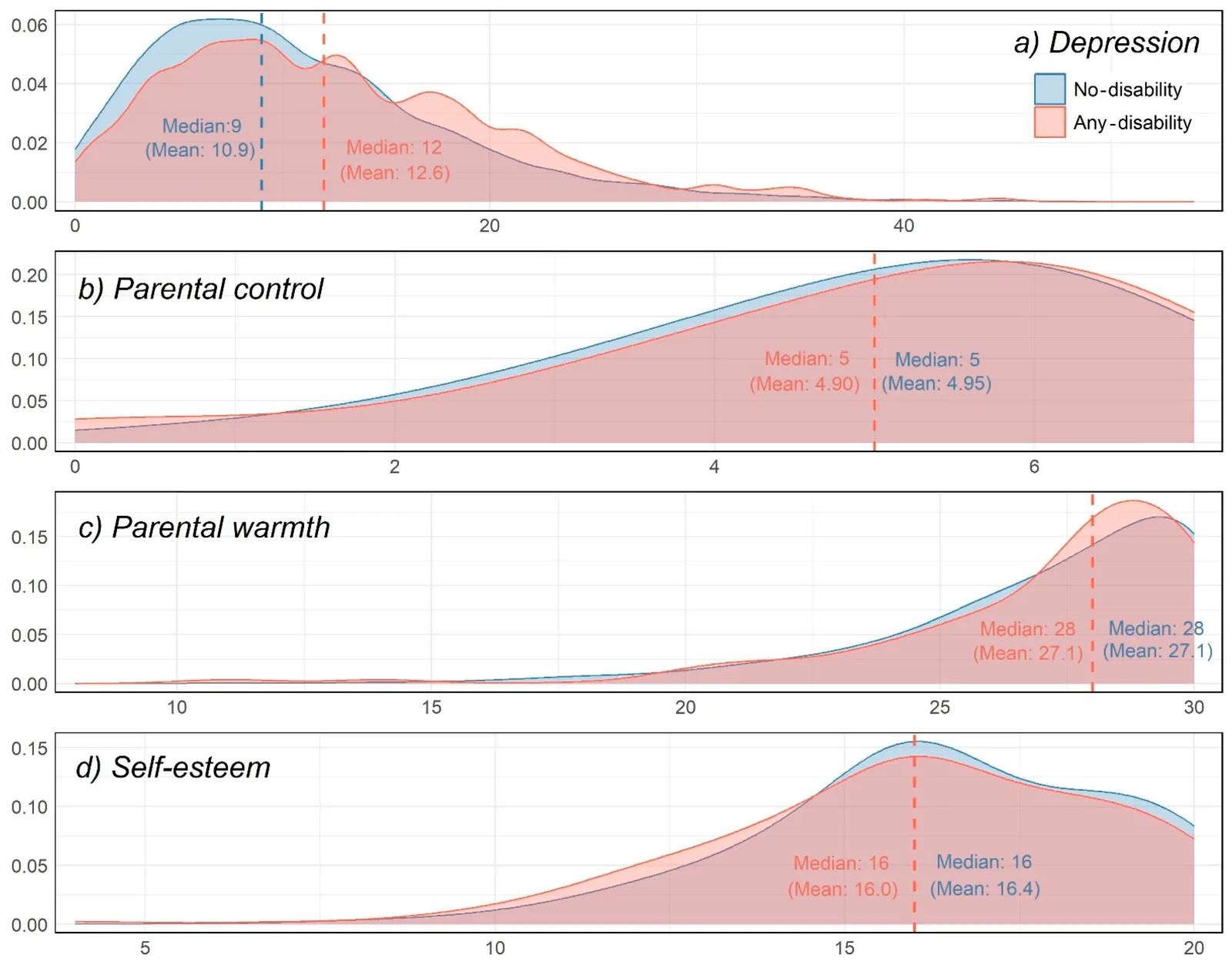
Density plots of the unstandardized psychological factors.

Figure 1 shows the density plot of the unstandardized psychological factors for individuals with and without disabilities. The any disability group (mean: 12.6) tended to have higher depressive symptom scores compared to the no disability group (mean: 10.9). Individuals without disabilities had higher self-esteem (mean: 16.4) than those with disabilities (mean: 16.0). The distribution was also slightly right-skewed, suggesting that higher self-esteem is more common among individuals without disabilities. For parental control and parental warmth, levels were similar between groups. Parental control averaged 4.90 in the any disability group and 4.95 in the no disability group, while parental warmth was identical in both groups (mean = 27.1), although its distribution differed slightly.

### 3.2. Psychological Factors and PA

We investigated the longitudinal associations of psychological factors with the changes in PA and SB levels during the transition from adolescence to young adulthood among individuals with and without disabilities. A total of sixteen statistical models were developed, eight for PA and eight for PI, by combining each psychological factor (depressive symptoms, parental control, parental warmth, and self-esteem) with disability status (no disability and any disability), while controlling for multiple demographic and socioeconomic variables.

A total of sixteen statistical models were developed, eight for PA and eight for SB, with separate models for each psychological factor (depressive symptoms, parental control, parental warmth, and self-esteem) within the no-disability and any-disability groups. As our primary focus is on psychological factors, we report only psychological factors below.

Among individuals without disabilities, each one-unit increase in depressive symptom scores was associated with a lower likelihood of decreasing physical activity (RRR = 0.32, 95% CI: 0.16–0.61) compared with maintaining stable levels of physical activity (Table 2). A similar inverse association was observed among individuals with disabilities (RRR = 0.14, 95% CI: 0.02–0.91). For increasing physical activity, depressive symptoms were not significantly associated with changes in physical activity among either individuals without disabilities (RRR = 1.65, 95% CI: 0.90–3.03) or those with disabilities (RRR = 0.81, 95% CI: 0.16–4.24).

Parental control and parental warmth showed opposite patterns of association with changes in physical activity. Among individuals without disabilities, each 1-SD increase in parental control scores was associated with a lower likelihood of decreasing physical activity (RRR = 0.68, 95% CI: 0.49–0.95) compared with maintaining stable levels. In contrast, each 1-SD increase in parental warmth scores was associated with a greater likelihood of decreasing physical activity (RRR = 2.60, 95% CI: 1.24–5.46) compared with maintaining stable levels.

Regarding self-esteem, each 1-SD increase in self-esteem scores was associated with a greater likelihood of decreasing physical activity (RRR = 4.38, 95% CI: 2.72–7.06) and a lower likelihood of increasing physical activity (RRR = 0.37, 95% CI: 0.23–0.60) among individuals without disabilities. Similar patterns were observed among individuals with disabilities, with each 1-SD increase in self-esteem associated with a greater likelihood of decreasing physical activity (RRR = 15.89, 95% CI: 2.11–119.53). Self-esteem was not significantly associated with increasing physical activity among individuals with disabilities (RRR = 1.01, 95% CI: 0.14–7.14). As Supplementary Information, we also report *p*-values, including false discovery rate-adjusted q-values, in Supplementary Table S12.

We also conducted pooled survey-weighted multinomial logistic regression analyses using the full analytic sample, including interaction terms between disability status and each psychological factor for PA. None of the interaction terms was statistically significant, indicating that the associations between the psychological factors and changes in PA did not significantly differ according to disability status. The results of these interaction analyses have been added to the Supplementary Material (Supplementary Table S13).

### 3.3. Psychological Factors and SB

Although most associations were not statistically significant, higher depressive symptom scores were associated with both decreasing and increasing trends in SB, particularly among adolescents with disabilities. Specifically, each 1-SD increase in depressive symptom scores was not significantly associated with decreasing SB among individuals without disabilities (RRR = 1.86, 95% CI: 0.82–4.20), but was associated with a higher likelihood of decreasing SB among individuals with disabilities (RRR = 19.08, 95% CI: 1.12–326.40) compared with maintaining stable levels (Table 3). Similarly, each 1-SD increase in depressive symptom scores was not significantly associated with increasing SB among individuals without disabilities (RRR = 2.14, 95% CI: 0.94–4.85), whereas it was associated with a higher likelihood of increasing SB among individuals with disabilities (RRR = 10.30, 95% CI: 1.19–89.07). In addition, among adolescents without disabilities, each 1-SD increase in parental warmth scores was not significantly associated with decreasing SB (RRR = 0.43, 95% CI: 0.17–1.11), but was associated with a lower likelihood of increasing SB (RRR = 0.35, 95% CI: 0.14–0.87) compared with maintaining stable levels. No statistically significant associations were observed among adolescents with disabilities. For self-esteem, no statistically significant associations were observed among adolescents without disabilities or those with disabilities. Among adolescents with disabilities, each 1-SD increase in self-esteem was associated with a lower likelihood of both decreasing SB (RRR = 0.08, 95% CI: 0.00–1.55) and increasing SB (RRR = 0.11, 95% CI: 0.01–1.07), although these associations did not reach statistical significance. As Supplementary Information, we also report *p*-values, including false discovery rate-adjusted q-values, in Supplementary Table S14. We also conducted pooled survey-weighted multinomial logistic regression analyses using the full analytic sample, incorporating interaction terms between disability status and each psychological factor to examine changes in SB. None of the interaction terms reached statistical significance, suggesting that the associations between the psychological factors and changes in SB did not significantly differ by disability status. The results of these interaction analyses have been added to the Supplementary Material (Supplementary Table S15).

## 4. Discussion

Previous research has rarely examined the role of psychological factors in shaping changes in PA and SB during the transition from adolescence to young adulthood among individuals with and without disabilities. This highlights the need to better understand how psychological determinants influence PA and SB over time, particularly in adolescents with and without disabilities, as this transitional period is critical phase for establishing lifelong health-related behaviors. Our study revealed several patterns in how these factors influence PA engagement and SB over time.

First, our findings suggest that psychological factors were associated with changes in PA, with some numerically larger associations observed among adolescents with disabilities. Although the interaction models did not identify statistically significant differences between disability groups, the direction and magnitude of several associations varied numerically across groups. In particular, depressive symptoms and self-esteem showed larger point estimates for some PA transitions among adolescents with disabilities, while parental warmth also showed a larger point estimate for decreasing PA. Nevertheless, confidence intervals overlapped across groups for several estimates, and not all associations reached statistical significance. These patterns may be considered in the context of prior studies indicating that youth with disabilities experience greater psychological vulnerability that may affect PA participation, particularly in the context of heightened personal and environmental barriers to PA participation [40,41]. We also found numerically stronger associations between changes in SB and depressive symptoms and self-esteem. Previous research has similarly highlighted greater psychological distress among individuals with disabilities and associations between psychological factors and SB [42]. Our results highlight the importance of managing psychological distress among individuals with disabilities. Interventions that promote PA and reduce SB, along with targeted mental health support, may help mitigate depressive symptoms and improve self-esteem in this population.

We observed contrasting associations of parental control and parental warmth among adolescents without disabilities. Specifically, higher parental control was linked to a reduced likelihood of decreased PA, while higher parental warmth was linked to an increased likelihood of decreased PA. Previous research on parenting factors and PA has reported mixed findings, suggesting that these associations may vary according to the specific dimensions of parenting, study design, and contextual factors [43,44,45,46,47,48,49]. Several factors may help contextualize our findings. First, most prior studies have focused on cross-sectional associations between parental warmth or parental control and PA, whereas our study examined changes in PA and SB from adolescence to young adulthood. Second, parental attitudes alone may be insufficient to fully explain their associations with PA and SB. For example, several studies have found that the influence of parental warmth on PA is moderated by factors such as the child’s gender and specific parenting practices [45]. Similarly, previous research has reported that children of permissive and authoritative parents tend to have higher levels of PA than those with authoritarian or uninvolved parents [46]. Moreover, expressions of warmth that involve overprotection or passive support may inadvertently hinder physical activity [47,48]. Experimental research with typically developing children also shows that active parental participation, rather than mere observation, drives increase in PA [49]. Our findings suggest that affective intent alone may be insufficient without corresponding behavioral follow-through.

In addition, among adolescents without disabilities, higher depressive symptoms were associated with a lower likelihood of decreasing PA, whereas higher self-esteem was associated with a greater likelihood of decreasing PA and a lower likelihood of increasing PA during the transition from adolescence to young adulthood. This pattern contrasts with cross-sectional studies suggesting that lower depressive symptoms [50] and higher self-esteem [51,52] are associated with greater PA participation. Several factors may explain this finding. First, adolescents with lower depressive symptoms and higher self-esteem may have already engaged in relatively high levels of PA during adolescence, leaving limited room for further increases, a phenomenon known as the ceiling effect [53,54]. Second, the decrease in PA among those with low depressive symptoms and high self-esteem may reflect the general downward trend in PA commonly observed during the transition to adulthood [55,56]. Third, adolescents with higher depressive symptoms and lower self-esteem may have shifted their motivation, using PA as a self-regulation strategy to cope with emotional stress during the emerging adulthood [40,57]. These findings suggest that interventions targeting youth with high depressive symptoms and low self-esteem may yield greater improvements in PA.

Finally, we observed that individuals with higher depressive symptoms were more likely to either decrease or increase their SB levels, regardless of disability status. Similarly, those with higher self-esteem were less likely to either decrease or increase their SB levels. These findings suggest that SB may be governed by other mechanisms, such as emotional regulation and behavioral stability, rather than physical capacity or access-related constraints, unlike PA [58]. For example, individuals with higher depressive symptoms may experience emotional dysregulation or fluctuations in motivation and energy, increasing the likelihood of changes in SB. In contrast, higher self-esteem may act as a protective factor, promoting stable daily routines and reducing substantial shifts in SB. The supplementary analyses revealed no statistically significant interaction effects by disability status. Although several associations with PA remained significant after FDR adjustment, none of the associations with SB remained significant. The mutually adjusted models also showed limited statistically significant associations when all four psychological factors were considered simultaneously. These findings suggest that the observed associations, particularly those involving SB, should be interpreted cautiously.

This study has several limitations. Given the longitudinal nature of the Add Health study, sample attrition between Waves I and IV and the exclusion of participants with incomplete data may have introduced selection bias. As a result, the representativeness of the analytic sample and the generalizability of the findings may have been affected. Psychological factors were assessed using self-reported survey items rather than clinical assessments and may therefore reflect transient emotional states rather than sustained affective conditions [59,60]. Baseline PA and SB levels were not separately accounted for in the analyses, which may have influenced the observed associations. Additionally, the timing of socioeconomic covariate measurements may not fully capture changes in socioeconomic circumstances between adolescence and young adulthood. In addition, PA and SB were assessed using different questionnaire structures in Waves I and IV, introducing uncertainty when interpreting longitudinal changes. Although normalization allowed the wave-specific measures to be expressed on a common numerical scale, it does not establish measurement equivalence between the Wave I and Wave IV instruments. Formal measurement invariance was not assessed. Min–max normalization may also be sensitive to extreme values and skewed distributions; therefore, the resulting longitudinal categories should be interpreted as relative patterns of change rather than direct changes measured using equivalent instruments.

The physical disability classification also represents a heterogeneous group of participants with potentially different types and levels of functional limitation; therefore, the findings should be interpreted as associations based on the broader binary classification used in this study rather than as patterns specific to particular disability types or severity levels. For contextual comparison, the weighted proportion of participants classified as having a physical disability in the present sample was 5.6%, compared with a contemporaneous national estimate of 6.7% for activity limitations associated with chronic conditions among U.S. children under age 18 [61]. However, these estimates are not directly comparable because of differences in age ranges and, importantly, the operational definitions of disability. The 5.6% observed in the present study reflects the specific Add Health-based classification used for the current analysis and should not be interpreted as a population prevalence estimate of physical disability among U.S. adolescents. Lastly, education and income were drawn from Wave IV, concurrently with the follow-up PA and SB measures. Their inclusion as covariates requires caution when interpreting the findings due to the temporal relationships between these factors and changes in PA/SB over time.

There are several opportunities for future research. Longitudinal and mixed-methods studies are needed to clarify the mechanisms through which psychological factors influence PA and SB trajectories over time, as our study design does not establish causal relationships. Additionally, contextual factors such as family dynamics, inclusive PA opportunities, self-regulation strategies, emotional regulation, and behavioral stability should be explored to explain variability in sensitivity to psychological influences across disability groups. These insights can inform the development of more targeted, sustainable public health strategies for promoting health-related behaviors across developmental stages and disability status.

## 5. Conclusions

This study examined the longitudinal associations between psychological factors, including depressive symptoms, parental control, parental warmth, and self-esteem, and changes in PA and SB during the transition from adolescence to young adulthood among individuals with and without physical disabilities. Our findings indicate that these psychological and parental factors were associated with different patterns of PA and SB change across the transition to young adulthood. The findings also highlight the importance of considering psychological and parental factors when examining PA and SB outcomes in both groups, while recognizing that the observed associations do not establish causal effects or differences in intervention effectiveness. Emotional closeness without behavioral follow-through may not necessarily promote activity, while associations involving parental control may vary depending on youth developmental needs and environmental contexts. Future research should examine whether interventions addressing psychological and parental factors, together with structured, accessible, and autonomy-supportive environments, can support PA participation during the transition to young adulthood.

## Figures and Tables

**Table 1:** Descriptive summary of participants using survey-weights.

Variables		No Disability (%)	Any Disability (%)
Unweighted sample size (n)		14,783	918
Weighted population estimate		17,521,176 (94.4)	1,037,186 (5.6)
Physical activity	Decrease (<−1 SD)	4,090,810 (23.4)	259,771 (25.1)
	Stable (−1 SD~1 SD)	10,710,655 (61.2)	631,172 (61.0)
	Increase (>1 SD)	2,704,185 (15.4)	143,861 (13.9)
Sedentary behavior	Decrease (<−1 SD)	1,825,232 (10.5)	80,372 (7.9)
	Stable (−1 SD~1 SD)	13,963,298 (80.4)	828,810 (81.7)
	Increase (>1 SD)	1,576,193 (9.1)	105,840 (10.4)

**Table 2:** Survey-weighted multinomial logistic regression results for changes in physical activity by psychological factors and disability status. Only relative risk ratios for psychological factors are reported to keep the table concise.

		Changes in Physical Activity	Relative Risk Ratio	95% Confidence Interval
Depressive symptoms	No disability	Decrease	0.32 *	**0.16~0.61**
		Increase	1.65	0.90~3.03
	Any disability	Decrease	0.14 *	**0.02~0.91**
		Increase	0.81	0.16~4.24
Parental control	No disability	Decrease	0.68 *	**0.49~0.95**
		Increase	0.98	0.62~1.56
	Any disability	Decrease	1.17	0.28~4.92
		Increase	1.89	0.47~7.68
Parental warmth	No disability	Decrease	2.60 *	**1.24~5.46**
		Increase	0.69	0.37~1.26
	Any disability	Decrease	3.30	0.34~31.76
		Increase	0.16	0.01~1.86
Self-esteem	No disability	Decrease	4.38 *	**2.72~7.06**
		Increase	0.37 *	**0.23~0.60**
	Any disability	Decrease	15.89 *	**2.11~119.53**
		Increase	1.01	0.14~7.14

Note: Bold values and asterisks (*) indicate statistically significant results (*p* < 0.05).

**Table 3:** Survey-weighted multinomial logistic regression results for changes in sedentary behavior by psychological factors and disability status. Only relative risk ratios for psychological factors are reported to keep the table concise.

		Changes in Sedentary Behavior	Relative Risk Ratio	95% Confidence Interval
Depressive symptoms	No disability	Decrease	1.86	0.82~4.20
		Increase	2.14	0.94~4.85
	Any disability	Decrease	19.08 *	**1.12~326.40**
		Increase	10.30 *	**1.19~89.07**
Parental control	No disability	Decrease	1.15	0.60~2.20
		Increase	0.96	0.58~1.59
	Any disability	Decrease	0.34	0.05~2.13
		Increase	0.50	0.12~2.14
Parental warmth	No disability	Decrease	0.43	0.17~1.11
		Increase	0.35 *	**0.14~0.87**
	Any disability	Decrease	0.12	0.00~5.20
		Increase	1.80	0.15~22.06
Self-esteem	No disability	Decrease	0.75	0.36~1.54
		Increase	0.55	0.26~1.18
	Any disability	Decrease	0.08	0.00~1.55
		Increase	0.11	0.01~1.07

Note: Bold values and asterisks (*) indicate statistically significant results (*p* < 0.05).

## Data Availability

The data used in this study were obtained under a data use agreement with the Add Health study administered by the Carolina Population Center at the University of North Carolina at Chapel Hill. All analyses were conducted within the Secure Research Workspace (SRW) and were approved for secondary analysis by the Add Health Data Disclosure Committee. Due to data security and confidentiality requirements, the Add Health data cannot be shared directly by the authors. Researchers may apply for access through the Add Health website (https://addhealth.cpc.unc.edu, accessed on 25 October 2026) under a formal data use agreement.

## Supplementary Materials

The following supporting information can be downloaded at https://www.mdpi.com/article/10.3390/healthcare14213733/s1. Figure S1: Flow diagram of participant selection and analytical sample sizes. Table S1: Descriptive statistics for the original, non-normalized PA and SB variables by wave among participants with disabilities. Table S2: Descriptive statistics for the original, non-normalized PA and SB variables by wave among participants without disabilities. Table S3: Depressive symptoms questions. Table S4: Parental control questions. Table S5: Parental warmth questions. Table S6: Self-esteem questions. Table S7: Correlations between psychological factors. Table S8: No disability with PA. Table S9: Any disability with PA. Table S10: No disability with SB. Table S11: Any disability with SB. Table S12: Relative risk ratios, *p*-values, and false discovery rate-adjusted q-values for changes in physical activity. Table S13: Interaction effects between disability status and psychological factors on changes in physical activity. Table S14: Relative risk ratios, *p*-values, and false discovery rate-adjusted q-values for changes in sedentary behavior. Table S15: Interaction effects between disability status and psychological factors on changes in sedentary behavior.

## Abbreviations

The following abbreviations are used in this manuscript:

PA	Physical Activity
SB	Sedentary Behavior
