# Supplementary material for: Associations Between Adolescent Psychological Factors and Physical Activity and Sedentary Behavior in Young Adulthood by Disability Status
Source: Healthcare (Basel). 2026 Nov 3;14(21):3733. doi: 10.3390/healthcare14213733 (PMC13763348; doi:10.3390/healthcare14213733)
Supplement: Supplementary file 1 [file healthcare-14-03733-s001.zip › healthcare-4507920-supplementary.pdf]

Supplementary Figure S1. Flow diagram of participant selection and analytical sample sizes.

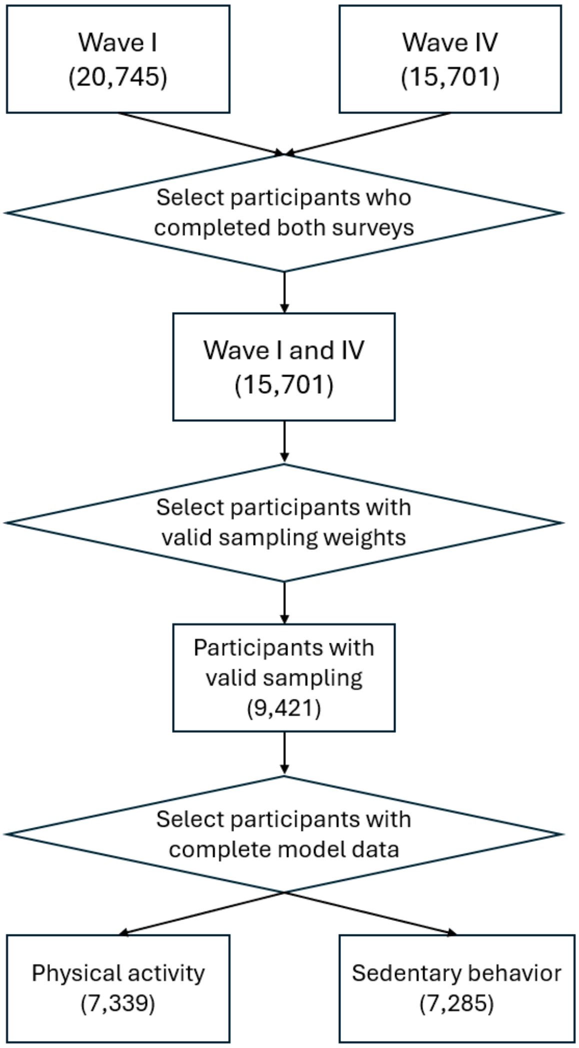

Supplementary Table S1. Descriptive statistics for the original, non-normalized PA and SB variables by wave among participants with disabilities.

		Mean	Median	Max	Min	SD
PA	Wave I	3.84	4	9	0	2.09
	Wave IV	21.27	17	90	2	15.34
SB	Wave I	22.51	16	200	0	21.36
	Wave IV	17.61	12	126	0	18.01

Supplementary Table S2. Descriptive statistics for the original, non-normalized PA and SB variables by wave among participants without disabilities.

		Mean	Median	Max	Min	SD
PA	Wave I	3.79	4	9	0	2.15
	Wave IV	21.82	18	110	0	15.52
SB	Wave I	22.96	17	282	0	21.34
	Wave IV	16.1	12	171	0	16.06

Supplementary Table S3. Depressive symptoms questions

Question text	Variable	Response value
How often was the following true during the past week?<br>You were bothered by things that don't usually bother you.	H1FS1	0: never or rarely<br>1: sometimes<br>2: a lot of the time<br>3: most of the time or all of the time
How often was the following true during the past week?<br>You didn't feel like eating, your appetite was poor.	H1FS2	
You felt that you could not shake off the blues, even with help from your family and your friends	H1FS3	
How often was the following true during the past week?<br>You felt you were just as good as other people.	H1FS4	
How often was the following true during the past week?<br>You had trouble keeping your mind on what you were doing.	H1FS5	
How often was the following true during the past week?<br>You felt depressed.	H1FS6	
How often was the following true during the past week?<br>You felt that you were too tired to do things.	H1FS7	
How often was the following true during the past week?<br>You felt hopeful about the future.	H1FS8	
How often was the following true during the past week?<br>You thought your life had been a failure.	H1FS9	
How often was the following true during the past week?<br>You felt fearful.	H1FS10	
How often was the following true during the past week?<br>You were happy.	H1FS11	
How often was the following true during the past week?<br>You talked less than usual.	H1FS12	
How often was the following true during the past week?<br>You felt lonely.	H1FS13	
How often was the following true during the past week?<br>People were unfriendly to you.	H1FS14	
How often was the following true during the past week?<br>You enjoyed life.	H1FS15	
How often was the following true during the past week?<br>You felt sad.	H1FS16	
How often was the following true during the past week?<br>You felt that people disliked you.	H1FS17	
How often was the following true during the past week?<br>It was hard to get started doing things.	H1FS18	
How often was the following true during the past week?<br>You felt life was not worth living.	H1FS19	

Supplementary Table S4. Parental control questions

Question text	Variable	Response value
Do your parents let you make your own decisions about the time you must be home on weekend nights?	H1WP1	Yes / No
Do your parents let you make your own decisions about the people you hang around with?	H1WP2	Yes / No
Do your parents let you make your own decisions about what you wear?	H1WP3	Yes / No
Do your parents let you make your own decisions about how much television you watch?	H1WP4	Yes / No
Do your parents let you make your own decisions about which televisio n programs you watch?	H1WP5	Yes / No
Do your parents let you make your own decisions about what time you go to bed on week nights?	H1WP6	Yes / No
Do your parents let you make your own decisions about what you eat?	H1WP7	Yes / No

Supplementary Table S5. Parental warmth questions

Question text	Variable	Response value
Do you agree or disagree with the following statement? Most o f the time, your mother is warm and loving toward you.	H1PF1	1: strongly agree<br>2: agree<br>3: neither agree nor disagree<br>4: disagree<br>5: Strongly disagree
How close do you feel to your {MOtheR/ADOPTIVE MOtheR/ STEPMOtheR/ FOSTER MOtheR/etc.}?	H1WP9	1: not at all<br>2: very little
How much do you think she [resident mother] cares about you?	H1WP10	3: somewhat<br>4: quite a bit<br>5: very much
Do you agree or disagree with the following statement? Most of the time, your father is warm and loving toward you.	H1PF23	1: strongly agree<br>2: agree<br>3: neither agree nor disagree<br>4: disagree<br>5: Strongly disagree
How close do you feel to your {FAtheR/ADOPTIVE FAtheR/ST EPFAtheR /FOSTER FAtheR/etc.}?	H1WP13	1: not at all<br>2: very little
How much do you think he [resident father] cares about you?	H1WP14	3: somewhat<br>4: quite a bit<br>5: very much

Supplementary Table S6. Self-esteem questions

Question text	Variable	Response value
Do you agree or disagree with the following statement?	H1PF30	
You have a lot of good qualities.		1: strongly agree
Do you agree or disagree with the following statement?	H1PF32	2: agree
You have a lot to be proud of.		3: neither agree nor
Do you agree or disagree with the following statement?	H1PF33	disagree
You like yourself just the way you are.		4: disagree
Do you agree or disagree with the following statement?	H1PF34	5: Strongly disagree
You feel like you are doing everything just about right.		

Supplementary Table S7. Correlations between psychological factors

	Parental control	Parental warmth	Self-esteem	Depressive symptoms
Parental control	-	-0.04	0.01	-0.04
Parental warmth	-	-	0.38	-0.36
Self-esteem	-	-	-	-0.47
Depressive symptoms	-	-	-	-

Supplementary Table S8. No disability with PA

	**Changes in physical activity**	**Relative risk ratio**	**p-value**
Depressive symptoms	Decrease	0.63	0.31
	Increase	1.19	0.70
Parental control	Decrease	0.78	0.20
	Increase	1.07	0.81
Parental warmth	Decrease	1.34	0.45
	Increase	0.87	0.72
Self-esteem	Decrease	2.93	0.00
	Increase	0.52	0.09

Supplementary Table S9. Any disability with PA

	**Changes in physical activity**	**Relative risk ratio**	**p-value**
Depressive symptoms	Decrease	0.14	0.10
	Increase	0.34	0.51
Parental control	Decrease	1.61	0.58
	Increase	0.87	0.93
Parental warmth	Decrease	1.67	0.69
	Increase	0.12	0.14
Self-esteem	Decrease	1.59	0.74
	Increase	0.82	0.91

Supplementary Table S10. No disability with SB

	Changes in physical activity	Relative risk ratio	p-value
Depressive symptoms	Decrease	2.60	0.18
	Increase	1.17	0.79
Parental control	Decrease	1.24	0.58
	Increase	0.74	0.34
Parental warmth	Decrease	0.54	0.25
	Increase	0.41	0.11
Self-esteem	Decrease	0.98	0.97
	Increase	0.73	0.55

Supplementary Table S11. Any disability with SB

	Changes in physical activity	Relative risk ratio	p-value
Depressive symptoms	Decrease	1.85	0.76
	Increase	3.01	0.50
Parental control	Decrease	0.92	0.97
	Increase	1.59	0.70
Parental warmth	Decrease	0.22	0.42
	Increase	3.29	0.44
Self-esteem	Decrease	0.28	0.64
	Increase	0.69	0.78

Supplementary Table S12. Relative risk ratios, p-values, and false discovery rate-adjusted q-values for changes in physical activity

		Changes in physical activity	Relative risk ratio	p-values	FDR-adjusted q-values
Depressive symptoms	No disability	Decrease	0.32	0.00	0.00
		Increase	1.65	0.10	0.14
	Any disability	Decrease	0.14	0.04	0.15
		Increase	0.81	0.80	0.96
Parental control	No disability	Decrease	0.68	0.02	0.05
		Increase	0.98	0.94	0.95
	Any disability	Decrease	1.17	0.83	0.94
		Increase	1.89	0.37	0.61
Parental warmth	No disability	Decrease	2.60	0.01	0.03
		Increase	0.69	0.22	0.28
	Any disability	Decrease	3.30	0.30	0.57
		Increase	0.16	0.14	0.37
Self-esteem	No disability	Decrease	4.38	0	0
		Increase	0.37	0	0
	Any disability	Decrease	15.89	0.01	0.03
		Increase	1.01	0.99	1.00

Supplementary Table S13. Interaction effects between disability status and psychological factors on changes in physical activity

Outcome	Psychological factor	PA level change	Disability RRR	Psychological Factor RRR	Interaction RRR
Physical activity	Depressive symptoms	Decrease	1.28 (0.40)	0.30 (0.00)	0.57 (0.65)
	Depressive symptoms	Increase	0.95 (0.87)	1.63 (0.12)	0.60 (0.63)
	Parental control	Decrease	0.89 (0.87)	0.67 (0.02)	1.50 (0.64)
	Parental control	Increase	0.42 (0.10)	0.98 (0.93)	2.62 (0.17)
	Parental warmth	Decrease	1.53 (0.74)	2.63 (0.01)	0.67 (0.79)
	Parental warmth	Increase	1.16 (0.90)	0.68 (0.21)	0.56 (0.66)
	Self esteem	Decrease	0.56 (0.50)	4.40 (0.00)	2.78 (0.36)
	Self esteem	Increase	0.56 (0.43)	0.39 (0.00)	1.66 (0.61)

Supplementary Table S14. Relative risk ratios, p-values, and false discovery rate-adjusted q-values for changes in sedentary behavior

		Changes in sedentary behavior	Relative risk ratio	p-values	FDR-adjusted q-values
Depressive symptoms	No disability	Decrease	1.86	0.14	0.17
		Increase	2.14	0.07	0.09
	Any disability	Decrease	19.08	0.04	0.13
		Increase	10.30	0.03	0.13
Parental control	No disability	Decrease	1.15	0.68	0.81
		Increase	0.96	0.88	0.94
	Any disability	Decrease	0.34	0.25	0.50
		Increase	0.50	0.35	0.57
Parental warmth	No disability	Decrease	0.43	0.08	0.13
		Increase	0.35	0.02	0.06
	Any disability	Decrease	0.12	0.27	0.42
		Increase	1.80	0.64	0.65
Self-esteem	No disability	Decrease	0.75	0.43	0.55
		Increase	0.55	0.12	0.17
	Any disability	Decrease	0.08	0.10	0.25
		Increase	0.11	0.06	0.23

Supplementary Table S15. Interaction effects between disability status and psychological factors on changes in sedentary behavior

Outcome	Psychological factor	PA level change	Disability RRR	Psychological Factor RRR	Interaction RRR
Sedentary behavior	Depressive symptoms	Decrease	0.43 (0.14)	1.99 (0.10)	7.29 (0.26)
	Depressive symptoms	Increase	0.66 (0.35)	2.20 (0.06)	5.16 (0.21)
	Parental control	Decrease	1.29 (0.72)	1.15 (0.67)	0.41 (0.34)
	Parental control	Increase	1.60 (0.43)	0.93 (0.77)	0.48 (0.37)
	Parental warmth	Decrease	0.41 (0.06)	0.89 (0.95)	0.59 (0.81)
	Parental warmth	Increase	0.35 (0.02)	0.21 (0.22)	6.58 (0.20)
	Self esteem	Decrease	2.05 (0.50)	0.73 (0.38)	0.23 (0.29)
	Self esteem	Increase	2.00 (0.42)	0.53 (0.10)	0.37 (0.41)
